# Propidium Monoazide-Treated, Cell-Direct, Quantitative PCR for Detecting Viable Chloramphenicol-Resistant *Escherichia coli* and *Corynebacterium glutamicum* Cells

**DOI:** 10.3390/genes14122135

**Published:** 2023-11-27

**Authors:** Yang Qin, Bo Qu, Bumkyu Lee

**Affiliations:** Department of Environment Science & Biotechnology, Jeonju University, Jeonju 55069, Republic of Korea; qinyang2013@jj.ac.kr (Y.Q.);

**Keywords:** genetically modified microorganisms, chloramphenicol-resistant, propidium monoazide treatment, quantitative PCR, viable cells detection

## Abstract

With the rapid development and commercialization of industrial genetically modified microorganisms (GMMs), public concerns regarding their potential effects are on the rise. It is imperative to promptly monitor the unintended release of viable GMMs into wastewater, the air, and the surrounding ecosystems to prevent the risk of horizontal gene transfer to native microorganisms. In this study, we have developed a method that combines propidium monoazide (PMA) with a dual-plex quantitative PCR (qPCR) approach based on TaqMan probes. This method targets the chloramphenicol-resistant gene (*CmR*) along with the endogenous genes D-1-deoxyxylulose 5-phosphate synthase (*dxs*) and chromosomal replication initiator protein (*dnaA*). It allows for the direct quantitative detection of viable genetically modified *Escherichia coli* and *Corynebacterium glutamicum* cells, eliminating the requirement for DNA isolation. The dual-plex qPCR targeting *CmR*/*dxs* and *CmR*/*dnaA* demonstrated excellent performance across various templates, including DNA, cultured cells, and PMA-treated cells. Repeatability and precision, defined as RSDr% and bias%, respectively, were calculated and found to fall within the acceptable limits specified by the European Network of GMO Laboratories (ENGL). Through PMA–qPCR assays, we determined the detection limits for viable chloramphenicol-resistant *E. coli* and *C. glutamicum* strains to be 20 and 51 cells, respectively, at a 95% confidence level. Notably, this method demonstrated superior sensitivity compared to Enzyme-Linked Immunosorbent Assay (ELISA), which has a detection limit exceeding 1000 viable cells for both GM bacterial strains. This approach offers the potential to accurately and efficiently detect viable cells of GMMs, providing a time-saving and cost-effective solution.

## 1. Introduction

The field of genetic engineering and molecular biology has advanced significantly, with its roots tracing back to the commercialization of the first genetically modified tomato, “Flavr Savr”, in 1994. This development has led to a substantial increase in the approval, commercialization, and cultivation of genetically modified crops worldwide over the past three decades [1]. Concurrently, the utilization of recombinant techniques in the development of genetically modified microorganisms (GMMs) has found broad applications across various sectors. These applications span agriculture, including biofertilizers; healthcare encompassing pharmaceuticals and immunogenic materials (antigens); bioremediation processes addressing environmental pollution control; as well as the production of food additives, enzymes for the food and feed industry, and various biorefinery sectors, such as fuels, chemicals, and energy [2,3,4]. Conducting a risk assessment for the release of all GMOs and their derived products is essential, especially given their applications in natural ecosystems, human health, and environmental remediation. However, specific concerns do arise regarding the accidental release of GMOs during commercial production, transport, and disposal in both open and closed systems. Of particular concern is the potential risk associated with the horizontal transfer of antimicrobial resistance (AMR) genes to pathogens, gut microbiota, and other microorganisms [5].

Hence, it is crucial to establish and enforce stringent biosafety management protocols within manufacturing facilities engaged in GMO production, covering plants, animals, and microorganisms, to diligently monitor and prevent any potential external emissions. For instance, it is important to consider the possible exposure during the contained use of GMMs on bioreactor surfaces, within airflow systems, and in wastewater, as well as the potential for contamination during activities such as sampling, inoculation, culture, or transfer between systems [6]. In such scenarios, the implementation of fast and effective monitoring and detection techniques becomes crucial for preventing GMM releases and mitigating potential risks. Furthermore, living genetically modified organisms (LMOs), especially LM microorganisms (LMMs), present an increased and inherently unpredictable risk to both human health and the environment, necessitating rapid identification and continuous monitoring.

Traditional GM testing techniques, like DNA-based semiquantitative polymerase chain reaction (PCR) and quantitative real-time PCR (qPCR), along with protein-based Enzyme-Linked Immunosorbent Assay (ELISA), have been employed to detect and quantify GMOs [7,8,9]. Fraiture et al. [10] employed PCR amplification of 16S rRNA sequences and the internal transcribed spacer (ITS) region to detect the presence of bacterial and fungal DNA. Subsequently, they implemented a combined qPCR approach targeting AMR genes, followed by viability testing and whole-genome sequencing. This investigation uncovered several unauthorized GMMs in food and feed products permitted for sale in the European Union market. Some of these GMMs were traced back to producing organisms like *Bacillus velezensis* and *Bacillus subtilis*, while others carried genetic material related to chloramphenicol (*CmR*), aminoglycoside adenyltransferase (*KanR*), and tetracycline (*TetR*) resistance genes [11,12,13]. Furthermore, the digital droplet PCR (ddPCR) technique was validated to provide precise quantification and enhanced sensitivity for detecting GMOs in food and environmental samples [14].

However, DNA-based GM detection methods have encountered specific limitations related to DNA isolation and quality. These challenges encompass the presence of PCR inhibitors originating from food matrices and chemical reagents, along with significant DNA loss [15,16]. Overcoming these hurdles often requires sophisticated techniques, labor, and considerable time to obtain substantial quantities of high-quality DNA. Direct PCR amplification systems have been successfully implemented across a wide range of organisms, including bacterial and fungal strains, various plant species, and human samples [17], but still struggle in qPCR analysis due to the presence of various matrix components acting as qPCR inhibitors. However, LMO detections often encounter false positives, which can result from DNA amplifications originating not only from living cells but also from dead cells or ejected DNA [18]. To mitigate this issue, the pretreatment of samples with nucleic acid intercalating dyes, such as propidium monoazide (PMA), has been suggested and successfully incorporated into both qPCR and ddPCR assays for viable cell detection within microbial populations in diverse matrices [19,20,21,22,23]. When it comes to the protein-based ELISA technique, there are potential bottlenecks in the quantification and sensitivity of GMM detection. Moreover, the task of distinguishing between living and dead cells based solely on protein levels presents a challenge, thereby restricting its widespread application in the detection of LMOs.

In general, microorganisms that have undergone comprehensive genetic, physiological, and biological characterization are typically the primary choice for industrial applications. Examples of such well-characterized microorganisms include the yeast *Saccharomyces cerevisiae*, the bacteria *Escherichia coli* (*E. coli*), *Corynebacterium glutamicum* (*C. glutamicum*), and filamentous fungi [2]. As of July 2023, the Republic of Korea has approved the commercial use of 20 GMMs spanning various industries. Among them, nine GMMs have been designed to utilize *C. glutamicum* as the host microorganism for producing L-arabinose isomerase, D-psicose-3-epimerase, D-tagatose 3-epimerase, D-fructose 4-epimerase, β-glucosidase, and 2’-fucosyllactose, intended for applications in the production of food materials. Additionally, six other GMMs, designed for applications in various industries, employ *E. coli* as the host microorganism [24]. Given the extensive use of *E. coli* and *C. glutamicum* strains in industrial GMMs, there is a pressing need to develop efficient methods for monitoring GMMs derived from these two hosts. In the current study, we have developed a TaqMan probe-based dual-plex qPCR method that utilizes cell-direct techniques, eliminating the need for DNA extraction. This approach is coupled with PMA treatment, enabling a rapid and efficient means of monitoring two chloramphenicol-resistant industrial LMMs, *E. coli* and *C. glutamicum*.

## 2. Materials and Methods

### 2.1. Bacteria Culture

Two GM bacteria used for the present study are a Gram-negative *E. coli* DH5α strain and a Gram-positive *C. glutamicum* ATCC13032 strain, with plasmid insertions of pACYC184 (4245 bp) and pXMJ19 (10,274 bp), respectively. Both strains harbor a chloramphenicol acetyltransferase gene (*CmR*) that confers resistance to chloramphenicol (Origin: pBR325). A single colony each of *E. coli* and *C. glutamicum* was individually introduced into 10 mL of Luria–Bertani (LB) broth (BD DifcoTM, Franklin Lakes, NJ, USA) and Luria–Bertani Brain–Heart Infusion (LBBHI) broth (BHI 10 g/L, BD DifcoTM, Franklin Lakes, NJ, USA), respectively. The media in both cases were supplemented with an antibiotic (25 μg/mL of chloramphenicol) and incubated for 18 h at 37 °C and 33 °C, respectively, with 180 rpm agitation.

### 2.2. Measurement of Optical Density and Colony-Forming Unit

The optical density at 600 nm (OD_600_) was determined in triplicate samples, and 1 mL of the 18 h cell culture was used for serial dilutions. A 10^−1^ diluted cell suspension was prepared by adding 100 µL of the 18 h cell culture to 900 µL of the LB or LBBHI broth. The same method was used to prepare further diluted cell suspensions ranging from 10^−2^ to 10^−8^. To minimize cell growth during experimental processes, all dilutions were carried out on ice. The serially diluted samples were subsequently utilized for both qPCR analysis and colony-forming unit (CFU) determination. Each 100 µL of 10^−6^, 10^−7^, and 10^−8^ diluted cell suspension was spread onto chloramphenicol-containing LB or LBBHI solid plates, and after overnight incubation, the average colony counts were calculated. CFU/mL was then determined using the following formula:

CFU/mL = average count number × 1/dilution rate × 1/inoculum, inoculum = 0.1 mL

The mean CFU/mL values of *E. coli* pACYC184 and *C. glutamicum* pXMJ19 strains were determined from 10 replications.

### 2.3. Primer and Probe Design for TaqMan-Based qPCR

We developed three specific primer sets: one targeting a chloramphenicol resistance gene (*CmR*), the second one targeting the single-copy endogenous taxon-specific gene D-1-deoxyxylulose 5-phosphate synthase gene (*dxs*, GenBank accession number AF035440) of *E.coli*, and the third one to detect the chromosomal replication initiator protein *dnaA* of *C. glutamicum* (NCBI reference sequence: NC_022198.1) for the detection of chloramphenicol-resistant bacterial strains pACYC184 and pXMJ19, respectively [25]. For the dual-plex qPCR assay, dual-labeled hydrolysis probes, including one of 5′-FAM/3′-BHQ1 for *CmR*, and two of 5′-HEX/3′-BHQ1 for the internal controls of *dxs* and *dnaA*, were designed as shown in Table 1.

### 2.4. DNA Extraction and Calibration

The genomic DNA was extracted from *E. coli* pACYC184 and *C. glutamicum* pXMJ19 using the Wizard^®^ Genomic DNA purification Kit (Promega, Madison, WI, USA), while plasmid DNA was extracted using the Wizard^®^ Plus Minipreps DNA purification system (Promega, Madison, WI, USA), according to the manufacturer’s instructions. The DNA concentration was determined using a NanoDrop spectrophotometer (MicroDigital Co., Ltd., Seongnam-si, Republic of Korea), and the purity was assessed by calculating the A260/A280 and A260/A230 ratios, where values >1.8 were considered indicative of high purity, and also by electrophoresis on 1.5% agarose gels.

To evaluate the performance of dual-plex qPCR assays for the detection of the combination of *CmR* and endogenous genes *dxs* or *dnaA* in two bacterial strains, *E. coli* pACYC184 and *C. glutamicum* pXMJ19, plasmid DNA and genomic DNA were used as DNA calibrants. In accordance with the methodology described by Whelan et al. [26], a range of DNA samples were serially diluted, spanning from 1 × 10^4^ to 1 × 10^8^ copies/µL. The samples consisted of both genomic and plasmid DNA and were prepared based on the lengths of the plasmids, 4245 bp and 10,274 bp for pACYC184 and pXMJ19, respectively, as well as the genome sequence lengths of 4,534,037 bp and 3,282,708 bp for *E. coli* [27] and *C. glutamicum* [28], respectively. Single-plex qPCR analysis was performed on plasmid DNA to obtain quantification cycle (Cq) values for the antibiotic resistance gene *CmR* at each dilution point. However, dual-plex qPCR analysis was used to determine the Cq value for each dilution point of the endogenous genes. A total of six qPCR runs were carried out in two intra-runs, each containing three replicates. Linear regression analysis was conducted using the mean Cq values of five diluted DNA samples, following standard curve generation. DNA copies were calculated by the formula as follows: DNA copies = 10^(Cq value − Y^_inter_^)/slope^. The amplification efficiency (E) of qPCR was determined by the slopes of each standard curve, as described by Rasmussen [29]. The repeatability and precision of the qPCR were assessed by calculating the relative standard deviation (RSDr%) and bias%. The bias% was determined using the following formula: bias% = (mean DNA copy − true DNA copy)/true DNA copy × 100.

### 2.5. qPCR Assay

The StepOne^TM^ Real-time PCR System (Applied Biosystems, Foster City, CA, USA) was used to perform both single-plex and dual-plex qPCR. Reactions were conducted in a final volume of 20 µL, comprising 10 µL of TOPreal™ qPCR 2X PreMIX (TaqMan Probe for multiplex, Enzynomics, Daejeon, Republic of Korea), 10 pmol of primers/probes, and 1 µL of DNA or 1 µL of homogenous cell suspension (kept on ice). Negative controls were set up using SDW and LB/LBBHI broth. The qPCR was conducted with the following parameters: 95 °C for 10 min, followed by 40 cycles of 95 °C for 30 s and 60 °C for 40 s. The abundance of target DNA was determined by calculating the Cq values, which represent the number of cycles needed to reach a predefined threshold value. Ten replications of qPCR intra-assays were conducted, with each consisting of three repeats inter-run. To verify the practicality of cell-direct qPCR and ensure the absence of matrix effects, cell suspensions were diluted 10-fold, ranging from 10^−1^ to 10^−6^, and subjected to qPCR amplification. The resulting Cq values at various dilution points were utilized to derive a linear regression equation to calculate cell-direct qPCR amplification efficiency.

### 2.6. Quantification of Viable Cells by PMA–qPCR Assay

To perform PMA treatment on each 400 µL of 18 h cell stock culture and its serially diluted *E. coli* or *C. glutamicum* cell suspensions (ranging from 10^−1^ to 10^−6^), 1 µL of 20 mM PMA (PMAxx™ Dye, Biotium Inc., Fremont, CA, USA) was added to make a working concentration of 25 uM of PMA solution. Then, we incubated tubes in the dark with 30 rpm of agitation at room temperature for ten minutes. The mixture was exposed to light to cross-link PMA to DNA on a PMA-Lite™ LED Photolysis Device (Biotium Inc., Fremont, CA, USA) for 15 min, then placed on ice and kept away from light until use. To perform the qPCR assays, we used the PMA-treated cells directly and followed the cell-direct qPCR conditions, with the only variation being a decreased primer/probe concentration of 5 pmol. Before use, each PMA-treated cell sample was mixed by vortex for 30 s to maintain a homogenous suspension. A total of six qPCR runs consisting of two qPCR intra-assays on PMA-treated cell suspensions were performed. Additionally, 12 PMA–qPCR runs were carried out on dilutions ranging from 10^−3^ to 10^−6^ and determined the limit of detection (LOD_95%_) using the Quodata web application [30] available at URL https://quodata.de/content/validation-qualitative-pcr-methods-single-laboratory (accessed on 26 March 2023). The viable cell counts were determined based on the average PMA-Cq values according to DNA standard curves, using the following formula, Viable cell counts/mL = 10^(PMA-Cq value − Y^_inter_^)/slope^ × dilution rate. The bias percentage was determined using the formula (Viable cell counts/mL of internal control − CFU/mL)/CFU/mL × 100, where CFU/mL was obtained from plate counting.

### 2.7. Statistical Analysis

Microsoft Excel was used for statistical analysis. The following parameters were analyzed: average values (Mean) and standard deviation (SD) of OD_600_, Cq, and PMA-Cq values obtained from the qPCR assay, along with CFU/mL determined through plate counting for the bacterial strains under investigation. Furthermore, a regression analysis was carried out on five DNA dilution points to construct DNA standard curves for the antibiotic gene *CmR*, as well as the endogenous genes *dxs* and *dnaA* of the two bacterial strains. Linear regression equations were generated by utilizing dynamic ranges of diluted cell and PMA-treated cell suspensions, and qPCR parameters, such as qPCR efficiency, slope, and R^2^, were determined.

## 3. Results and Discussion

### 3.1. qPCR Performance and DNA Standard Curve Construction

#### 3.1.1. Specificity of Primer and Probe

To confirm the specificity of three target sequences (*CmR*, *dxs*, and *dnaA*), in silico analysis was performed using the basic local alignment search tool (BLAST) server at the National Center for Biotechnology Information (NCBI). This assay involved comparing the expected PCR amplification sequences generated by each primer set, and the results validated the specificity of the three target sequences. Furthermore, PCR amplifications were performed using genomic DNA from two bacterial strains, and the results were consistent with the in silico analysis. The amplification products confirmed the presence of a specific 116 bp fragment corresponding to *CmR* in both bacterial strains. Additionally, a specific band of 160 bp for *dxs* was observed in the *E. coli* strain, while a band of 196 bp for *dnaA* was detected in the *C. glutamicum* strain. Sterile distilled water (SDW) was used as a negative control, and no amplification was observed. In addition, dual-plex qPCR was conducted for both bacterial strains. The results indicated that the negative controls (SDW) exhibited Cq values exceeding 35 for both *CmR*/*dxs* and *CmR*/*dnaA*, in comparison to the Cq values of 15.03/15.44 and 15.06/15.16, respectively, obtained from the genomic DNA of *E. coli* and *C. glutamicum*, respectively (Figure S1). Therefore, the primer and probe sets employed in the dual-plex qPCR assay exhibited specificity for detecting both bacterial strains.

#### 3.1.2. qPCR Parameters

**Figure 1:**
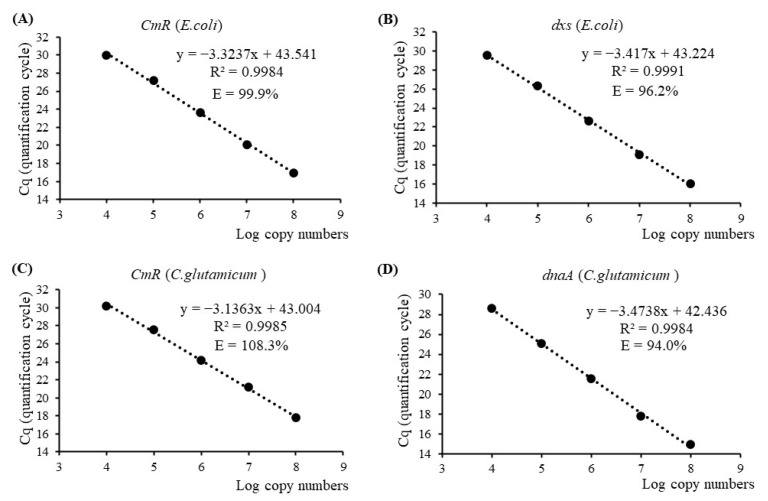
DNA standard curves for the antibiotic-resistant gene *CmR* and taxon-specific endogenous genes *dxs* and *dnaA* of *E. coli* and *C. glutamicum*, respectively, using single-plex or dual-plex quantitative real-time PCR (qPCR) analysis. E—qPCR efficiency; R^2^—linear correlation coefficient. (**A**) *CmR* standard curve for *E. coli*; (**B**) *dxs* standard curve for *E. coli*; (**C**) *CmR* standard curve for *C. glutamicum*; (**D**) *dnaA* standard curve for *C. glutamicum*.

Standard curves were constructed for each target gene using five dilution points ranging from 1 × 10^4^ to 1 × 10^8^ copies of plasmid and/or genomic DNA isolated from the two bacterial strains. For the *CmR* gene of *E. coli* and *C. glutamicum*, regression equations were generated with DNA quantities ranging from 46.5 femtograms (fg) to 465 picograms (pg) and 113 fg to 1.13 nanograms (ng), respectively (Table S1). The resulting qPCR efficiencies were calculated to be 99.9% and 108.3%, with slopes of −3.3237 and −3.1363, respectively (Figure 1A,C). Regarding the endogenous genes *dxs* and *dnaA*, dual-plex qPCR exhibited satisfactory performance in regression analysis using genomic DNA quantities ranging from 50 pg to 500 ng for the *E. coli* strain and 36 pg to 360 ng for the *C. glutamicum* strain. The slopes of the regression lines were determined to be −3.417 and −3.4738, corresponding to qPCR efficiencies of 96.2% and 94.0%, respectively (Figure 1B,D). All four standard curves demonstrated strong linearity, as evidenced by coefficients of determination (R-squared values) greater than 0.99. Furthermore, the repeatability and accuracy of qPCR, typically assessed using two parameters, namely RSDr% and bias%, exhibited ranges of 0.15 to 1.85 and −19.54 to 22.94, respectively, for all dilution points forming four standard curves. These values fell within the allowable limits of ±25%, as specified in the guidelines provided by the European Network of GMO Laboratories (ENGL) [31]. These results suggested that dual-plex qPCR assays using *CmR*/*dxs* and *CmR*/*dnaA* were effective in detecting chloramphenicol-resistant *E. coli* and *C. glutamicum* strains, respectively, excluding nonspecific cross-reactivity of primer/probe combinations and potential effects of dimer formation.

### 3.2. Viable GM Bacterial Cells Detection by PMA-Treated Cell-Direct qPCR

#### 3.2.1. Performance of Cell-Direct qPCR Amplification on Two GM Bacterial Strains

According to the performance of DNA-based dual-plex qPCR assays targeting *CmR*/*dxs* and *CmR*/*dnaA*, we performed cell-direct qPCR analysis instead of DNA extractions. Following an 18 h cell culture of 10 replicates, we obtained average OD_600_ values of 0.77 ± 0.06 and 0.79 ± 0.05, corresponding to mean CFU/mL values of 2.18 ± 0.82 × 10^9^ and 1.46 ± 0.30 × 10^9^ by plate counting, respectively, for the *E. coli* and *C. glutamicum* strains. During the cultivation of the *C. glutamicum* strain, we observed that its growth did not consistently surpass an OD600 value of 0.70 within the designated 18 h period. This phenomenon was attributed to an extended lag phase evident in specific individual colonies. To ensure uniformity, we selected 18 h cell cultures that exceeded the 0.70 threshold for subsequent CFU and qPCR analyses. Moreover, previous research has confirmed that utilizing matrix substances such as LB broth as dilution substrates does not exhibit any noticeable adverse effects on the efficacy of the cell-direct dual-plex qPCR approach when performed on *KmR*/*dxs* and *nptII*/*dxs* genes [32]. This study also observed consistent outcomes, with Cq values of 35.49/35.43 for LB broth and 35.76/36.35 for SDW targeting *CmR*/*dxs*, 36.24/37.06 for LBBHI broth, and 36.21/36.78 for SDW targeting *CmR*/*dnaA* (Table S2).

**Figure 2:**
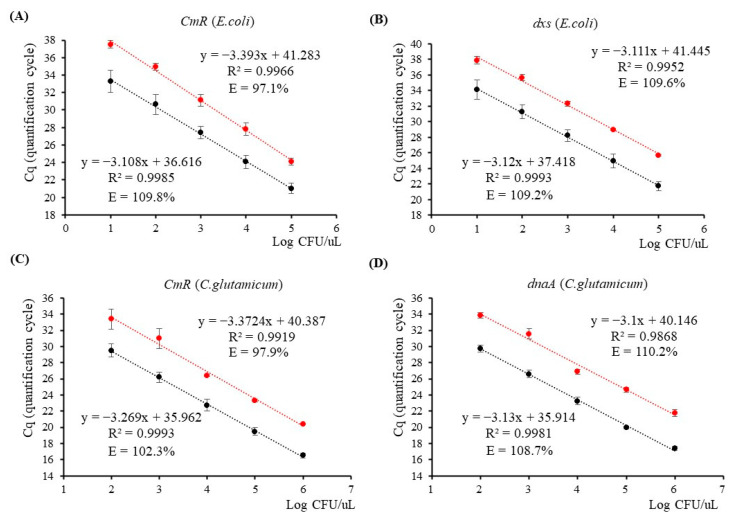
Quantitative PCR assays targeting *CmR*/*dxs* and *CmR*/*dnaA* genes using cell-direct dual-plex and propidium monoazide (PMA)-treated qPCR methods. (**A**,**B**) *CmR* and *dxs* qPCR assays were used to detect genetically modified *E. coli* cells in serially diluted cell and PMA-treated cell suspensions, respectively; (**C**,**D**) *CmR* and *dnaA* qPCR assays were used to detect genetically modified *C. glutamicum* cells in serially diluted cell and PMA-treated cell suspensions, respectively. Black dots indicate the average Cq values of diluted cell points; red dots indicate the average Cq values of PMA-treated diluted cell points.

The cell-direct qPCR analysis was conducted using the 18 h cell culture and its five serially diluted suspensions ranging from 10^−1^ to 10^−5^, and the linear regression equation was established using the resulting Cq values, as shown in Figure 2. For *the E. coli* strain, the 18 h cell culture yielded Cq values of 19.31 ± 0.23 for *CmR* and 20.29 ± 0.26 for *dxs* (Table 2). The qPCR efficiencies for amplification of *CmR* and *dxs* were found to be 109.8% and 109.2%, respectively, with corresponding slopes of −3.108 and −3.12 (as shown in Figure 2A,B). As for the *C. glutamicum* strain, the Cq values obtained from the 18 h cell culture were 16.52 ± 0.29 for *CmR* and 17.40 ± 0.29 for *dnaA* (Table 2). Linear regression equations were constructed using five data points, including the 18 h stock cell culture and four diluted samples ranging from 10^−1^ to 10^−4^. The qPCR amplification efficiencies of *CmR* and *dnaA* were calculated to be 102.3% and 108.7%, respectively, with corresponding slopes of −3.269 and −3.13 (Figure 2C,D). All four linear regression lines exhibited strong correlation coefficients greater than 0.99. Furthermore, the calculation of RSDr% for the Cq values obtained from all dilution points targeting *CmR*/*dxs* and *CmR*/*dnaA* yielded a range of 1.20 to 3.73 and 0.87 to 3.83, respectively (Table S2). These measurements remained within the permissible ±25% range stipulated by ENGL, signifying a notable level of qPCR repeatability. These findings indicate a consistent distribution of cell density within each diluted suspension of both *E. coli* and *C. glutamicum*, with a 10-fold reduction in each instance. In addition to viable and nonviable cells, the cell culture also contains cell debris and various metabolic waste products. Our results reveal minimal matrix effects in 1 μL for qPCR, as confirmed by well-defined qPCR curves targeting *CmR*/*dxs* and *CmR*/*dnaA*, as shown in Figure S2A,B. Sung and Hawkins [33] conducted research on a TaqMan-based real-time PCR assay for cell culture medium, eliminating the need for DNA extraction, and successfully detected fewer than 10 CFU of mycoplasma contaminants in mammalian cell cultures. Furthermore, prior studies investigating cell-direct qPCR analysis of Kanamycin-resistant GM *E. coli* reported comparable results [32]. In contrast to DNA-based techniques, the utilization of TaqMan-based cell-direct dual-plex qPCR in this study demonstrated exceptional effectiveness in accurately detecting and quantifying both chloramphenicol-resistant *E. coli* and *C. glutamicum* cells.

#### 3.2.2. Identification of Viable Cells Using PMA-Treated Cell-Direct qPCR

To identify viable bacterial cells, 18 h cell cultures and their diluted cell suspensions were treated with PMA. Subsequently, dual-plex qPCR analysis on the PMA-treated cell suspensions was conducted, and the results demonstrated delayed Cq values in comparison to the untreated samples, for both bacterial strains, when targeting the CmR gene as well as the endogenous genes *dxs* and *dnaA* (Figure S2C,D). For the *E. coli* strain, the Cq values for both targets were consistently delayed by 3 cycles across five diluted cell suspensions. This observation suggests that the DNA quantities derived from viable cells in the samples account for only a tenth (1/10) of the total proportion of cell DNA quantities (Figure 2A,B, Table S2). However, it is worth noting that a slight delay in the Cq values of the PMA-treated 18 h cell cultures, particularly when targeting CmR, could potentially be attributed to the inhibitory effect of higher cell concentrations on qPCR amplification. By employing the PMA-treated cell-direct qPCR analysis method, the expected Cq values for detecting 218,000 viable cells (corresponding to 10^−1^ dilution) are 24.12 ± 0.41 for the CmR and 25.68 ± 0.07 for the *dxs* (Table S2). We performed linear regression analysis on five dilution points of PMA-treated *E. coli* cells for both target genes. The regression equations exhibited strong correlation coefficients, resulting in qPCR efficiencies of 97.1% for CmR and 109.6% for *dxs*.

Regarding the *C. glutamicum* strain, the Cq values obtained through dual-plex qPCR analysis for detecting 1,460,000 viable *C. glutamicum* cells were found to be 20.38 ± 0.11 for the *CmR* gene and 21.79 ± 0.43 for the *dnaA* gene after PMA treatment (Table 2). A comparison of the Cq values before PMA treatment revealed noticeable delays of approximately 3 cycles for the PMA-treated 18 h culture and its diluted samples (10^−1^ to 10^−4^). Consequently, linear regression equations were derived, exhibiting expected slopes and strong correlation coefficients (Figure 2C,D). The efficiencies of PMA–qPCR targeting the *CmR* and *dnaA* genes were calculated to be 97.9% and 110.2%, respectively. The repeatability of qPCR for both bacterial strains was evaluated by calculating the RSDr% for each dilution point, and the results were found to fall within the acceptable range of ±25%, as specified by ENGLs (Table S2).

PMA, acting as a photosensitive dye, possesses the ability to specifically enter dead cells with compromised membrane integrity, subsequently cross-linking with their DNA. This cross-linking process effectively hinders the amplification during PCR [18,34]. Conventionally, samples treated with PMA are subjected to DNA extraction prior to PCR analysis, ensuring that PMA does not interfere with qPCR efficiency as a PCR inhibitor [21]. Joo et al. [35] reported limitations in their attempts to distinguish viable phytoplankton cells using PMA. They found that PMA treatment reduced DNA yield from live cells by as much as 54.9% compared to untreated live cells. In our experiment, however, PMA-treated cells were employed directly. This approach not only serves to label damaged cells but also introduces the possibility of residual PMA acting as a potential PCR inhibitor, thereby impeding the performance of qPCR. To tackle this issue, the cell culture was diluted to a range of 10-fold (10^−1^) to 100,000-fold (10^−5^), and then each suspension was treated with 20 mM of PMA solution. Moreover, in the qPCR assays, we employed half the concentration of primers and probes to mitigate PCR inhibitions, a strategy that had demonstrated notable efficacy in a prior study [32]. As a result, the performance of qPCR on treated cells at each dilution point can be verified (Figure S2C,D). PMA-treated cells consistently exhibited lower delta Rn values than untreated cells when targeting specific genes. This suggests the possibility that PMA, as one of the fluorescence signals, contributes to an increase in passive reference dye levels, ultimately resulting in a reduced delta Rn reading. Another potential factor to consider could be the use of half-probe concentrations, which may lead to relatively lower fluorescence signals. Nevertheless, the curve shapes and uniform distances across the diluted samples indicate that there is no significant inhibition of qPCR.

The resulting Cq values from the PMA–qPCR were then used for subsequent linear regression analysis. The ENGL has defined acceptance criteria for qPCR detection of GMOs in DNA samples. These criteria involve a slope range between −3.1 and −3.6, which corresponds to qPCR amplification efficiencies of 110% to 90% in the absence of inhibition. However, when dealing with overprocessed food or feed samples, a slope within the range of −4.1 to −3.1 is considered acceptable [31]. In the case of the *E. coli* strain, the regression equations yielded permissible PMA–qPCR efficiencies when targeting *CmR*/*dxs*. As for the *C. glutamicum* strain, the PMA–qPCR efficiency when targeting *CmR* falls within the acceptable range, but when targeting *dnaA*, the efficiency exceeds 110%. When constructing linear regression equations with four data points, encompassing the 18 h cell culture and three dilution points down to 10^−3^, the PMA–qPCR amplification efficiency of *dnaA* is determined to be 105.7%, indicating that it falls within the acceptable range. This finding implies that within the 10^−4^ diluted *C. glutamicum* cell suspension, where there is a limited amount of DNA available as the qPCR template, the residual PMA solutions function as inhibitors for PCR.

#### 3.2.3. Quantifying Viable Cell Counts Using PMA–qPCR Assay

Considering the presence of multi-copy plasmid DNA, viable cell counts for cultured *E. coli* and *C. glutamicum* strains were determined by averaging the Cq values acquired from PMA–qPCR assays targeting endogenous genes. Notably, the *dxs* gene in *E. coli* and the *dnaA* gene in *C. glutamicum* cells each existed as a single copy. This computation relied on the DNA standard curves generated. Furthermore, the average Cq values were derived from four dilution points from 10^−1^ to 10^−4^ of cell cultures. This approach was adopted to account for the potential presence of inhibitors in concentrated samples (18 h cell cultures). The resulting viable cell counts per milliliter were calculated to be 1.93 × 10^9^ for *E. coli* and 1.69 × 10^9^ for *C. glutamicum*, as detailed in Table 2. To confirm these results, we utilized plate counting techniques to ascertain the actual viable cell numbers (defined as CFU/mL). The findings revealed values of 2.18 × 10^9^ for *E. coli* and 1.46 × 10^9^ for *C. glutamicum* per milliliter. The bias% between the viable cell count obtained from the qPCR assay and the true viable cell numbers from plate counting was calculated to be −11.75% and 15.93%, respectively. These values fall within the acceptable trueness range of ±25% as defined by ENGLs. These outcomes emphasize the accuracy of the cell-direct PMA–qPCR assay in effectively identifying viable, genetically modified bacterial cells.

Moreover, plasmid copy numbers within the two bacterial strains were ascertained by calculating the ratio of cell counts resistant to chloramphenicol (*CmR*) to the cell counts associated with the endogenous genes. Upon utilizing genomic DNA as the qPCR template, the calculated plasmid copy numbers were 2.45 for the *E. coli* strain and 7.53 for the *C. glutamicum* strain. Employing the cell-direct qPCR approach led to plasmid copy numbers of 2.79 and 8.21 for *E. coli* and *C. glutamicum*, respectively. However, subsequent treatment with PMA resulted in adjusted plasmid copy numbers of 2.93 for *E. coli* and 8.67 for *C. glutamicum* (as shown in Table 2). These findings indicated a range of 2 to 3 plasmid copies in the *E. coli* strain and 7 to 8 plasmid copies in the *C. glutamicum* strain. Consequently, the qPCR assay utilizing cell suspensions and PMA-treated cell suspensions demonstrated consistent copy numbers when compared to DNA-based qPCR analysis.

### 3.3. Sensitivity to Detect Viable Bacterial Cells via PMA–qPCR

The PMA-treated cell-direct dual-plex qPCR assays, targeting *CmR*/*dxs* and *CmR*/*dnaA*, were conducted with 18 replicates for each, spanning four diluted cell suspensions ranging from 10^−3^ to 10^−6^ of the two bacterial strains, with the purpose of assessing the detection limit of viable bacterial cells. As a reference, PMA-treated LB broth and LBBHI broth were included in each qPCR run, serving as negative controls. The average Cq values for *CmR*/*dxs* were 37.07/39.04, while for *CmR*/*dnaA*, they were 35.30/36.21 (Table S2). We determined the percentage of positive runs in relation to the total runs, considering the Cq values below the negative control for both target genes. A percentage exceeding a 95% confidence threshold was used as the detection limit. As indicated in Table 3, both strains were detectable down to a 10,000-fold (10^−4^) dilution, which corresponds to an average of 218 viable *E. coli* cells and 146 viable *C. glutamicum* cells. We additionally utilized the Quodata web tool to conduct a more detailed analysis of the limit of detection at a 95% confidence level (LOD_95%_). The findings indicated that the cell-direct PMA–qPCR method could reliably detect a minimum of 20 viable chloramphenicol-resistant *E. coli* cells and 51 viable *C. glutamicum* cells, with a confidence interval ranging from 11 to 35 and 29 to 91, respectively (Figure S3).

We also conducted Enzyme-Linked Immunosorbent Assay (ELISA) on both GM bacteria strains to identify viable cells resistant to chloramphenicol within cell cultures. Among 10^9^ viable cells, the chloramphenicol levels were found to be 1848.2 ± 79.9 nM per milliliter for the *E. coli* strain and 2093.5 ± 503.9 nM per milliliter for the *C. glutamicum* strain. Notably, there was a higher expression of chloramphenicol in the *C. glutamicum* cells compared to the *E. coli* cells. In addition, there were significant deviations in chloramphenicol content among repetitions of the *C. glutamicum* strains. Donovan et al. [36] investigated the growth and division of these strains and revealed distinctive behavior in *C. glutamicum* compared to *E. coli* and *B. subtilis*. This differentiation arose due to the absence of functional homologs in the Min and nucleoid occlusion (Noc) systems, leading to unequal cell division and the production of unequally sized daughter cells. In our own experimentation, we observed that 18 h cell cultures exhibited varying CFU and OD_600_ values. To ensure consistency, we selected cultures with similar characteristics for both the qPCR assay and ELISA. However, the divergent chloramphenicol content detected via ELISA from identical viable cells indicated the presence of dissimilar proportions of dead cells intermixed with the viable ones.

In order to establish a discernible limit of detection for ELISA, a range of viable cells from 10^8^ to 10^5^ were prepared for assessing chloramphenicol content. As depicted in Figure S4, chloramphenicol detection remained feasible down to 10^6^ viable cells (equivalent to 10^3^ per microliter), resulting in concentrations of 3.9 ± 1.6 nM and 26.6 ± 7.3 nM/mL for *E. coli* and *C. glutamicum*, respectively. With *E. coli* cells, a 10-fold reduction in chloramphenicol content was observed with each dilution. Conversely, the chloramphenicol content of *C. glutamicum* cells at 10^9^ and 10^8^ did not exhibit the same 10-fold discrepancy, likely due to the limitations of the ELISA kit’s measurement range, spanning only from 0.37 nM to 1500 nM. Furthermore, the quantification of chloramphenicol content encompassed cumulative levels not just from viable chloramphenicol-resistant bacterial cells but also from nonviable ones. As a result, the detection limit of ELISA should be established to exceed 1000 viable cells per microliter. Therefore, the detection sensitivity of ELISA for viable LM *E. coli* and *C. glutamicum* cells was found to be inferior to that of the PMA–qPCR assay, with a minimum detection threshold of 20 *E. coli* cells and 51 *C. glutamicum* cells per microliter.

Utilizing the cell-direct PMA–qPCR assay and the associated regression equations allows efficient identification of viable chloramphenicol-resistant *E. coli* and *C. glutamicum* cells that may have been unintentionally released into various environments, including wastewater, waste, airflow, and interior surfaces of large-scale food, feed, and bioreactor industries. Importantly, this detection can be achieved without complex DNA extraction procedures. Moreover, the combination of the chloramphenicol-resistant gene with endogenous genes in PCR can effectively differentiate various GM bacterial strains and determine their cell counts. This approach helps prevent false positives, such as the presence of naturally occurring chloramphenicol-resistant microorganisms in the surrounding environment. However, its applicability may be limited when handling LM microorganisms in environments with high inhibitor levels, such as humic acid in soil. In such cases, it may become essential to concentrate samples through procedures such as filtration or pretreatment before initiating the detection process.

## 4. Conclusions

As industrial genetically modified microorganisms (GMMs) are developed and commercialized, the need for rapid and efficient detection methods becomes crucial to monitor the presence of living GMMs (LMMs) unintendedly released into the surrounding environment. In this study, PMA treatment, in combination with a dual-plex TaqMan-based qPCR method targeting the AMR gene *CmR* and the taxon-specific gene *dxs* or *dnaA*, was developed to detect viable LM *E. coli* or *C. glutamicum* cells. Both qPCR and PMA–qPCR assays were conducted on diluted cell suspensions for both target genes, demonstrating acceptable levels of sensitivity and accuracy, falling within the ±25% limit specified in the ENGL guidelines. Furthermore, the qPCR analysis remained unaffected by the cell culture matrix and the presence of minimal amounts of residue PMA solution. The PMA–qPCR assay was successful in achieving a detection limit with 95% confidence, capable of identifying as few as 20 viable chloramphenicol-resistant *E. coli* cells and 51 viable *C. glutamicum* cells. This performance surpasses that of the Enzyme-Linked Immunosorbent Assay (ELISA), which has a detection limit of 1000 viable chloramphenicol-resistant cells of both the *E. coli* and *C. glutamicum* strains. Notably, this method eliminates the need for high-level technical requirements associated with DNA extraction and purification, resulting in time and cost savings. Additionally, it prevents false positive detections in LMM cells and excludes the presence of natural chloramphenicol-resistant microorganisms. This research presents a practical and effective approach for monitoring the presence of LMMs in environmental samples, addressing an increasingly important concern in various industries.

## Figures and Tables

**Table 1:** Primer/probe sequences of targeting antibiotic resistance genes and their taxon-specific endogenous genes.

Target Genes	Direction	Primer/Probe Sequence	Size (bp)
Chloramphenicol resistance (*CmR*)	CmR-F	5′-TTCTGCCGACATGGAAGCCA-3′	116
	CmR-R	5′-CTTCTTCGCCCCCGTTTTCAC-3′	
	CmR-P	5′-FAM-AACCTGAATCGCCAGCGGCATCAGCACC-BHQ1-3′	
D-1-deoxyxylulose 5-phosphate synthase (*dxs*)	dxs-F	5′-AAGGCATTGTGAAGCGTCGT-3′	160
	dxs-R	5′-CTGGCGGCCATTTCCAGAAT-3′	
	dxs-R	5′-Hex-CGCTGAACGCCACGCTGGTCG-BHQ1-3′	
Chromosomal replication initiator protein (*dnaA*)	dnaA-F	5′-ATTGAAAACGATTTGGGCGATGC-3′	196
	dnaA-R	5′-CGACCACCAACTTCATACTGCT-3′	
	dnaA-P	5′-Hex-CGCTGCGCATGGGCCGATCATTCAGC-BHQ1-3′	

**Table 2:** Viable cell counts and quantification of plasmid copy number for two genetically modified bacterial strains.

Bacterial Species	Targets	qPCR (gDNA)	Plasmid Copy Number (DNA)	qPCR (Cell)	Plasmid Copy Number (Cell)	PMA–qPCR (Cell)	Viable Cell Count/mL (×10^9^)	Plasmid Copy Number (PMA-Cell)	CFU/mL (×10^9^)	Bias (%)
*E. coli* (pACYC184)	*CmR*	15.03 ± 0.18	2.45	19.31 ± 0.23	2.79	19.74 ± 0.25	7.11 ± 3.44	2.93	2.18 ± 0.04	−11.75
	*dxs*	15.44 ± 0.20		20.29 ± 0.26		22.29 ± 0.03	1.93 ± 0.85			
*C. glutamicum* (pXMJ19)	*CmR*	15.06 ± 0.10	7.53	16.52 ± 0.29	8.21	20.38 ± 0.11	14.67 ± 4.89	8.67	1.46 ± 0.36	15.93
	*dnaA*	15.16 ± 0.07		17.40 ± 0.29		21.79 ± 0.43	1.69 ± 0.73			

**Table 3:** Limit of detection (LOD) for viable genetically modified bacterial cells using PMA–qPCR assay.

Bacterial Species	Targets	Cell Stock (18 h Culture)	10^−1^	10^−2^	10^−3^	10^−4^	10^−5^	10^−6^	LOD_95%_
*E. coli* (pACYC184)	CFU/uL	2,180,000	218,000	21,800	2180	218	21.8	2.18	20 (11–35)
	*dxs*	6/6 (100%)	6/6 (100%)	6/6 (100%)	18/18 (100%)	18/18 (100%)	17/18 (94.4%)	6/18 (33.3%)	
	*CmR*	6/6 (100%)	6/6 (100%)	6/6 (100%)	18/18 (100%)	18/18 (100%)	16/18 (88.9%)	9/18 (50%)	
*C. glutamicum* (pXMJ19)	CFU/uL	1,460,000	146,000	14,600	1460	146	14.6	1.46	51 (29–91)
	*dnaA*	6/6 (100%)	6/6 (100%)	6/6 (100%)	18/18 (100%)	18/18 (100%)	10/18 (55.6%)	2/18 (11.1%)	
	*CmR*	6/6 (100%)	6/6 (100%)	6/6 (100%)	18/18 (100%)	18/18 (100%)	10/18 (55.6%)	5/18 (27.8%)	

## Data Availability

The datasets used and/or analyzed within the frame of the present study can be provided by the corresponding author upon a justified request.

## Supplementary Materials

The following supporting information can be downloaded at: https://www.mdpi.com/article/10.3390/genes14122135/s1, Table S1: Repeatability and precision of dual-plex qPCR assay for chloramphenicol-resistant gene (*CmR*) and taxon-specific genes *dxs* for *Escherichia coli* and *dnaA* for *Corynebacterium glutamicum* using serial dilutions of genome DNA from two cell strains; Table S2: Cell-direct and PMA-treated cell-direct dual-plex qPCR performance at serially diluted points; Figure S1: Dual-plex qPCR using genome DNA of genetically modified *Escherichia coli* (*E. coli*) and *Corynebacterium glutamicum* (*C. glutamicum*) strains to verify the specificity of primer and probe combinations. (A) qPCR for *E. coli* targeting *CmR*/*dxs*; (B) qPCR for *C. glutamicum* targeting *CmR*/*dnaA*; sterile distilled water (SDW) was used as a negative control; Figure S2: Dual-plex qPCR data: utilizing both untreated cell and PMA-treated cell, was assessed following dilution targeting *CmR*/*dxs* and *CmR*/*dnaA* for two LM bacterial strains *E. coli* and *C. glutamicum*. (A) cell-direct qPCR for *E. coli* targeting *CmR*/*dxs*; (B) cell-direct qPCR for *C. glutamicum* targeting *CmR*/*dnaA*; (C) PMA-treated cell-direct qPCR for *E. coli* targeting *CmR*/*dxs*; (D) PMA-treated cell-direct qPCR for *C. glutamicum* targeting *CmR*/*dnaA*; Figure S3: Plausibility check of limit of detection (LOD) at 95% confidence interval for viable *E. coli* and *C. glutamicum* cells harboring *CmR*-resistant genes analyzed by the Quodata web application; (A) *E. coli* cells; (B) *C. glutamicum* cells; Figure S4: Detecting the chloramphenicol concentrations released from viable genetically modified bacterial cells through Enzyme-Linked Immunosorbent Assay (ELISA).
