# Supplementary material for: Propidium Monoazide-Treated, Cell-Direct, Quantitative PCR for Detecting Viable Chloramphenicol-Resistant Escherichia coli and Corynebacterium glutamicum Cells
Source: Genes (Basel). 2023 Nov 27;14(12):2135. doi: 10.3390/genes14122135 (PMC10743000; doi:10.3390/genes14122135)
Supplement: Supplementary file 1 [file genes-14-02135-s001.zip › genes-2722152-supplementary.pdf]

Supplementary Material

1 Supplementary Data

1.1 Materials and methods

Protein extraction and Enzyme-Linked Immunosorbent Assay

The proteins from two bacterial strains were extracted using 1XPBS buffer (137 mM NaCl, 2.7 mM KCl, 8 mM Na2HPO4, 2 mM KH2PO4, pH 7.4) based on the protocol for extracting chloramphenicol from cells, which was adapted from the chloramphenicol competitive ELISA kit (Cell Biolabs, Inc., San Diego, CA, USA). Briefly, 1×10^9, 1×10^8, 1×10^7, 1×10^6 and 1×10^5 cells (based on CFU/mL of 18 h culture cells) were respectively centrifuged at 13,000 rpm at 4 °C for 10 min, and then the supernatants were removed, the pellet was resuspended in 1 mL of cold PBS by vortexing for 30 s. The tubes containing cell suspensions were maintained on ice, and the cells were homogenized ten times for 2 seconds each, with a 1-second interval between each homogenization, respectively. After the homogenization, the tubes were left on ice for 30 minutes. The supernatant was centrifuged at 4 °C for 10 min at 13,000 rpm and then transferred to a new 1.5 ml tube. All samples were stored at –80 °C till further analysis.

To quantify chloramphenicol concentrations, Enzyme-Linked Immunosorbent Assay (ELISA) was performed on prepared protein samples using the supported technical protocol. Chloramphenicol standards were prepared for each experiment, with dilution series in the concentration range of 0, 0.37, 1.5, 6, 23, 94, 375 and 1500 nM /mL. The ELISA plate was read for absorbance at 450nm using a Multiskan SkyHigh microplate spectrophotometer (Thermo Fisher Scientific, Waltham, MA, USA). The samples were prepared in two replicates and three separate ELISAs were performed, and the average chloramphenicol concentrations were calculated.

2 Supplementary Figures and Tables

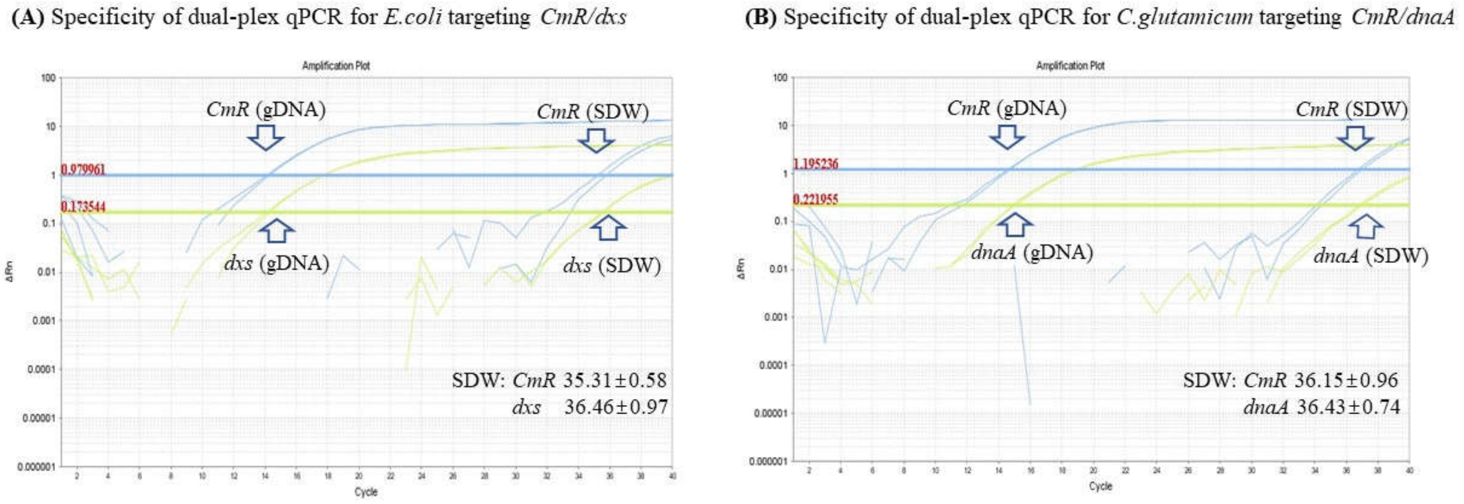

Figure S1. Dual-plex qPCR using genome DNA of genetically modified *Escherichia coli* (*E.coli*) and *Corynebacterium glutamicum* (*C.glutamicum*) strain to verify specificity of primer and probe combinations. (A) qPCR for *E.coli* targeting *CmR*/*dxs*; (B) qPCR for *C.glutamicum* targeting *CmR*/*dnaA*; Sterile distilled water (SDW) was used as negative control.

Table S1. Repeatability and precision of dual-plex qPCR assay for chloramphenicol resistant gene (*CmR*) and taxon-specific genes *dxs* for *Escheri chia coli* and *dnaA* for *Corynebacterium glutamicum* using serial dilutions of genome DNA from two cell strains.

Species	Targets	True copy	Mean Cq	Mean copy	SD	RSDr %	Bias %	Total DNAs (ng)
Escherichia coli (pACYC184)	*CmR* (plasmid DNA)	100000000	16.95	99866153	0.09	0.50	-0.13	0.465
		10000000	20.12	11104820	0.33	1.62	11.05	0.0465
		1000000	23.66	955873	0.40	1.67	-4.41	0.00465
		100000	27.24	80472	0.51	1.85	-19.53	0.000465
		10000	30.02	11732	0.12	0.39	17.32	0.0000465
	dxs (genomic DNA)	100000000	16.05	89658195	0.06	0.38	-10.34	500
		10000000	19.11	11404273	0.06	0.32	14.04	50
		1000000	22.61	1078394	0.05	0.23	7.84	5
		100000	26.30	89719	0.08	0.30	-10.28	0.5
		10000	29.54	10108	0.09	0.30	1.08	0.05
Corynebacterium glutamicum (pXJM19)	*CmR* (plasmid DNA)	100000000	17.77	111445317	0.03	0.18	11.45	1.13
		10000000	21.17	9169469	0.06	0.29	-8.31	0.113
		1000000	24.17	1010851	0.04	0.18	1.09	0.0113
		100000	27.58	82592	0.06	0.21	-17.41	0.00113
		10000	30.24	11743	0.05	0.15	17.43	0.000113
	dnaA (genomic DNA)	100000000	14.92	80463953	0.07	0.46	-19.54	360
		10000000	17.79	12294394	0.11	0.64	22.94	36
		1000000	21.52	1067311	0.15	0.68	6.73	3.60
		100000	25.11	102234	0.10	0.41	2.23	0.36
		10000	28.63	10165	0.12	0.42	1.65	0.036

Table S2. Cell-direct and PMA-treated cell-direct dual-plex qPCR performance at serial diluted points.

Bacterial species	Serial dilution	Cell-direct qPCR performance				PMA treated cell-direct qPCR performance			
	Targets	*CmR*	RSDr%	*dxs*	RSDr%	*CmR*	RSDr%	*dxs*	RSDr%
Escherichia coli (pACYC184)	Cell culture (18h)	19.31±0.23	1.20	20.29±0.26	1.28	19.74±0.25	1.29	22.29±0.03	0.14
	10^{-1}	21.03±0.61	2.88	21.71±0.34	1.57	24.12±0.41	1.69	25.68±0.07	0.27
	10^{-2}	24.07±0.77	3.22	24.95±0.65	2.61	27.82±0.74	2.65	29.00±0.14	0.48
	10^{-3}	27.43±0.69	2.51	28.21±0.53	1.88	31.11±0.70	2.24	32.31±0.32	1.00
	10^{-4}	30.65±1.14	3.71	31.27±0.94	3.01	34.95±0.43	1.22	35.67±0.38	1.07
	10^{-5}	33.28±1.24	3.73	34.15±0.62	1.82	37.52±0.42	1.12	37.90±0.48	1.27
Negative control (matrix)	LB	35.49±0.14	0.39	35.43±1.01	2.84	37.07±1.34	3.63	39.04±0.40	1.01
	SDW	35.76±0.66	1.83	36.35±0.53	1.45	-	-	-	-
	Targets	*CmR*	RSDr%	*dnaA*	RSDr%	*CmR*	RSDr%	*dnaA*	RSDr%
Corynebacterium glutamicum (pXJM19)	Cell culture (18h)	16.52±0.29	1.73	17.40±0.29	1.66	20.38±0.11	0.54	21.79 ±0.43	1.97
	10^{-1}	19.47±0.46	2.36	19.98±0.17	0.87	23.31±0.18	0.76	24.65 ±0.28	1.12
	10^{-2}	22.75±0.70	3.07	23.26±0.45	1.94	26.40±0.09	0.35	27.57 ±0.30	1.12
	10^{-3}	26.18±0.66	2.53	26.58±0.47	1.78	30.99±0.08	0.27	31.55 ±0.65	2.06
	10^{-4}	29.51±0.84	2.84	29.75±0.42	1.41	33.40±0.06	0.17	33.84 ±0.37	1.09
	10^{-5}	32.56±1.25	3.83	32.84±0.97	2.95	34.70±0.13	0.39	34.85 ±0.16	0.45
Negative control (matrix)	LBBHI	36.24±0.89	2.45	37.06±1.19	3.22	35.30±0.22	0.62	36.21 ±0.09	0.24
	SDW	36.21±0.56	1.55	36.78±0.71	1.92	-	-	-	-

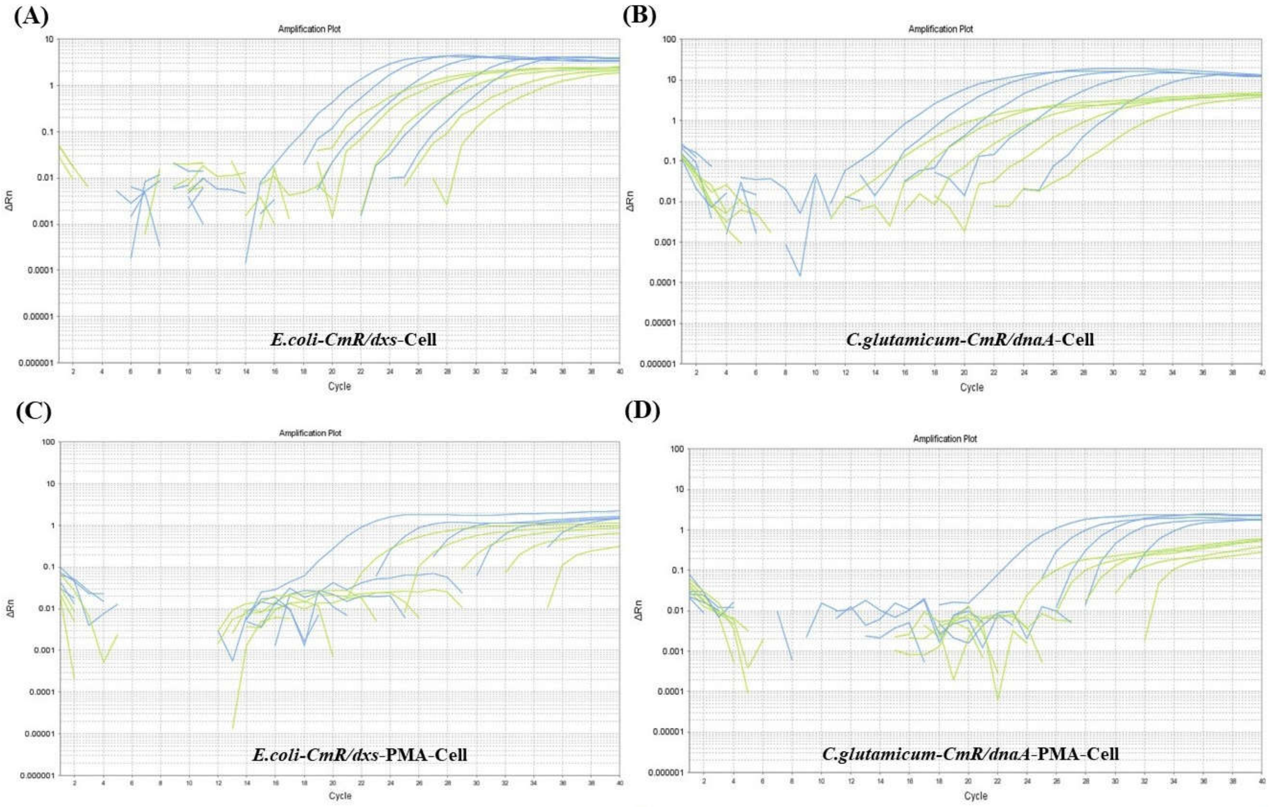

Figure S2. Dual-plex qPCR data: utilizing both untreated cell and PMA-treated cell, was assessed following dilution targeting *CmR*/*dxs* and *CmR*/*dnaA* for two LM bacterial strains *E.coli* and *C.glutamicum*. (A) cell-direct qPCR for *E.coli* targeting *CmR*/*dxs*; (B) cell-direct qPCR for *C.glutamicum* targeting *CmR*/*dnaA*; (C) PMA-treated cell-direct qPCR for *E.coli* targeting *CmR*/*dxs*; (D) PMA-treated cell-direct qPCR for *C.glutamicum* targeting *CmR*/*dnaA*.

(A) LOD detection for chloramphenicol resistant *E.coli* cells

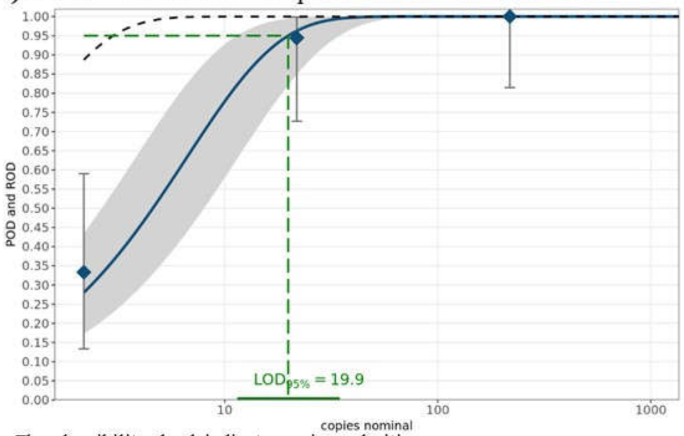

The plausibility check indicates no irregularities.
POD curve and LOD95%
The LOD95% is 19.860 with a 95 % confidence interval of [11.460, 34.646].
The figure below summarises the results. The blue diamonds characterise the laboratory-specific RODs. The blue curve denotes the mean POD curve along with the corresponding 95 % confidence range highlighted as the grey band. The POD curve under ideal conditions is displayed as the black dashed curve.

(B) LOD detection for chloramphenicol resistant *C.glutamicum* cells

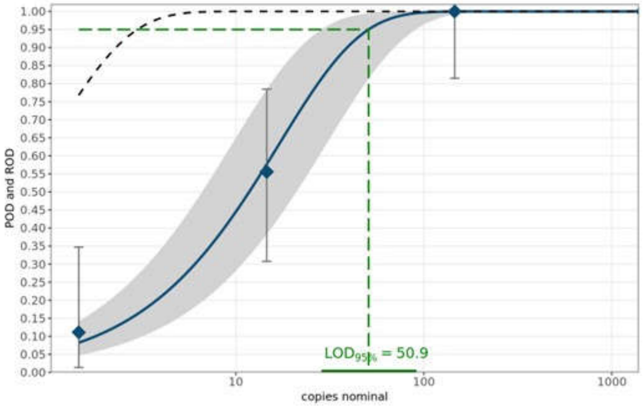

The plausibility check indicates no irregularities.
POD curve and LOD95%
The $LOD_{95\%}$ is 50.873 with a 95 % confidence interval of [28.553, 91.232].
The figure below summarises the results. The blue diamonds characterise the laboratory-specific RODs. The blue curve denotes the mean POD curve along with the corresponding 95 % confidence range highlighted as the grey band. The POD curve under ideal conditions is displayed as the black dashed curve.

Figure S3. Plausibility check of limit of detection (LOD) at 95% confidence interval for viable *E.coli* and *C.glutamicum* cells harboring *CmR*- resi stant genes analyzed by Quodata web application; (A) *E.coli* cells; (B) *C.glutamicum* cells.

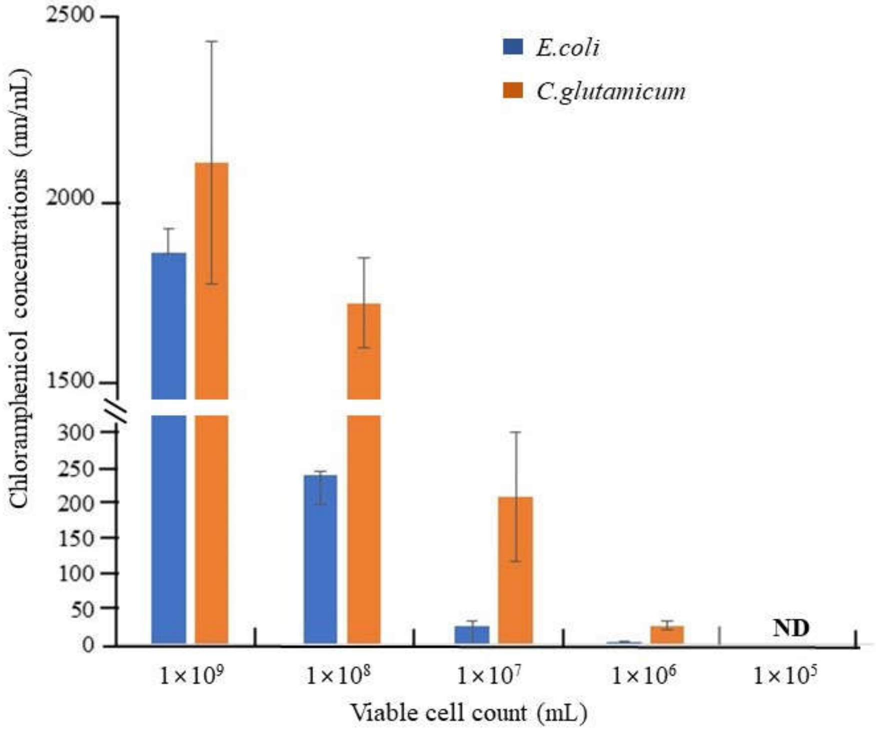

Figure S4. Detecting the chloramphenicol concentrations released from viable genetically modified bacterial cells through Enzyme-Linked Immun osorbent Assay (ELISA).
